# Identifying molecular markers suitable for *Frl* selection in tomato breeding

**DOI:** 10.1007/s00122-018-3136-0

**Published:** 2018-07-07

**Authors:** Zübeyir Devran, Erdem Kahveci, Yiguo Hong, David J. Studholme, Mahmut Tör

**Affiliations:** 1 Faculty of Agriculture, Department of Plant Protection, University of Akdeniz, Antalya, Turkey; 2 Department of Plant Pathology, M.Y. Genetik Agriculture Technology Laboratory, Antalya, Turkey; 3 College of Life and Environmental Sciences, Research Centre for Plant RNA Signaling, Hangzhou Normal University, Hangzhou, China; 4 Biosciences, College of Life and Environmental Sciences, University of Exeter, Exeter, UK; 5 Institute of Science and the Environment, University of Worcester, Worcester, WR2 6AJ UK

## Abstract

Modern plant breeding heavily relies on the use of molecular markers. In recent years, next generation sequencing (NGS) emerged as a powerful technology to discover DNA sequence polymorphisms and generate molecular markers very rapidly and cost effectively, accelerating the plant breeding programmes. A single dominant locus, *Frl*, in tomato provides resistance to the fungal pathogen *Fusarium oxysporum* f. sp. *radicis*-*lycopersici (FORL)*, causative agent of *Fusarium* crown and root rot. In this study, we describe the generation of molecular markers associated with the *Frl* locus. An F_2_ mapping population between an *FORL* resistant and a susceptible cultivar was generated. NGS technology was then used to sequence the genomes of a susceptible and a resistant parent as well the genomes of bulked resistant and susceptible F_2_ lines. We zoomed into the *Frl* locus and mapped the locus to a 900 kb interval on chromosome 9. Polymorphic single-nucleotide polymorphisms (SNPs) within the interval were identified and markers co-segregating with the resistant phenotype were generated. Some of these markers were tested successfully with commercial tomato varieties indicating that they can be used for marker-assisted selection in large-scale breeding programmes.

**Electronic supplementary material:**

The online version of this article (10.1007/s00122-018-3136-0) contains supplementary material, which is available to authorized users.

## Introduction

Cultivated tomato (*Solanum lycopersicum* L.) is the second most important consumed vegetables, grown worldwide and cultivated for fresh market and processed consumption (Foolad and Panthee 2012). As with many other crop plants, many pests and pathogens including viruses, nematodes, bacteria and fungi attack cultivated tomato varieties. Tomato vascular wilts and crown and root rot diseases are the most important devastating diseases and are caused by the fungal pathogens *Fusarium oxysporum f. sp. lycopersici (FOL)* and *Fusarium oxysporum f. sp. radicis*-*lycopersici (FORL)*, respectively (Lievens et al. 2009). *FORL* has been reported to have a broader host range than *FOL* (Edel-Hermann et al. 2011) and is considered to cause significant yield losses in the greenhouse, open field crops and soilless production system. Although *FORL* is a soil-borne pathogen, dissemination of air-borne microconidia helps pathogen to re-infect the plants and cause an epidemic (Szczechura et al. 2013).

Several approaches to control *FORL* have been taken. These include: (a) pesticide applications such as using carbendazim (MBC) and the soil fumigant dazomet (DAZ) (Zhao et al. 2016); (b) use of biopesticides or biological control agents such as *Bacillus* species (Baysal et al. 2013); (c) soil solarization where pathogen infested soil is covered with polythene cover and the soil is subjected to high solar temperature (Saremi et al. 2008); and (d) use of resistant varieties such as those that contain the *Frl* gene (Fazio et al. 1999).

A comparison of these various control methods shows that the use of resistant varieties is highly desirable, cost-effective and environmentally safe. A genetic locus designated *Frl* provides resistance to *FORL* in tomato. Originally this locus was introduced into the cultivated *S. lycopersicum* from the wild species *S. peruvianum* (Fazio et al. 1999) and since then, it has been used in commercial plant breeding programmes.

Transcriptomics, proteomics and genetics studies have been performed to understand *FORL*-tomato interactions especially with tomato lines carrying *Frl* locus. Transcriptomic studies carried out with resistant and susceptible near isogenic lines revealed that in incompatible interactions, defence genes related to secondary metabolites and tryptophan metabolism showed elevated expression level, while in compatible interactions, increased level of gene expression related to oxidative burst and necrosis have been observed (Manzo et al. 2016).

Mazzeo et al. (2014) took a differential proteomic approach to investigate changes in resistant and susceptible tomato cultivars infected with *FORL*. Their results showed that there is accumulation of defence-related proteins including glutathione S-transferase in the resistant cultivar while proteins involved in redox reactions accumulated in susceptible cultivar.

Genetic studies showed that *Frl* is a single dominant locus on chromosome 9 and several molecular markers including the RFLP-based marker TG101 have been shown to be linked to *Frl* (Truong et al. 2011). As thousands of plant lines are screened in a given breeding programmes, marker-assisted selection (MAS) offers advantages over classic phenotype-based selection. This is due to the fact that it can save time, resources and efforts and selection can be done at the seedling stages (Collard and Mackill 2008). Further molecular studies resulted in the development of RAPD (Truong et al. 2011) and SCAR (Mutlu et al. 2015) markers that are linked to the *Frl*. We have tried some of these markers but none of these have proven to be close enough to be successfully used in high throughput breeding programmes as they are several cM away from the *Frl* locus allowing the high number of recombination events to occur, which is not desirable for commercial breeding activities.

Here, we used next-generation sequencing technology and bulk segregant analysis method to develop markers that are tightly linked to *Frl*, MAS-friendly, easy to use and reliable. We report the generation of these markers and demonstrate that they can clearly separate the resistant and susceptible lines even in the available commercial lines.

## Materials and methods

### Plant lines and mapping populations

An F_2_ mapping population was generated from a cross between the susceptible (MT-7000) and resistant (MT-7028) tomato pure lines by breeders (Multi Tohum A.Ş., Antalya, Turkey) and was used in the experiments.

### Fungal isolate and pathology methods

*Fusarium oxysporum* f.sp. *radicis*-*lycopersici* isolate, No: F-125 (M.Y. Genetik Tarım Teknoloji Laboratuvar Tic. Ltd. Şti, Antalya, Turkey), was maintained in 50% glycerol at − 80 °C and was used throughout this study. The fungus was grown in Czapek Dox Broth on a rotary shaker for 7 days at 23 °C. The broth culture was filtered through two layers of cheesecloth. The suspension was centrifuged at 3750 rpm for 15 min and conidia were re-suspended in sterile dH_2_0. The roots of seedlings at one true leaf stage were washed off substrate, dipped in a suspension of 10^7^ spores/ml. Seedlings were then transplanted in a sterilized mixture and kept in a growth chamber at 23 °C with a 12 h photoperiods for 30 days. Control plants were treated with sterile dH_2_0 in a similar manner. At the end of incubation period, plants were evaluated as resistant or susceptible on the basis of existence of brown lesions on roots and crowns (Fazio et al. 1999; Xu et al. 2006).

### DNA isolation and genome sequencing

Young leaves were collected from plants after pathotyping with *FORL*. Plant genomic DNA was then isolated using the Wizard Magnetic Kit (Promega) following the manufacturer’s instructions. DNA was extracted separately from each individual of the F_2_ plant lines. The resistant and susceptible bulks were generated from twenty resistant and twenty susceptible F_2_ individuals, respectively, as described (Devran et al. 2015) and used for genomic sequencing. We generated 1 lane of 100 bp paired-end Illumina HiSeq 2500 sequencing data for each parent (resistant and susceptible) line and bulked (resistant and susceptible) pools.

### Bioinformatics and NGS analysis

The Illumina reads were first trimmed based on their quality scores using BBDuk (filterk = 27, trimk = 27; http://jgi.doe.gov/data-and-tools/bb-tools/) to remove Illumina adapters and to quality–trim both ends to Q12. *Frl* was mapped previously on tomato chromosome 9 (https://solgenomics.net/locus/566/view). We then used trimmed sequences from resistant and susceptible parents and resistant and susceptible bulks on the reference genome using Burrows-Wheeler Alignment tool (BWA) (Li and Durbin 2009). The region on chromosome 9:4205900–5108100 was extracted using SAMtools (Li et al. 2009) (https://sourceforge.net/projects/samtools/files/), and single-nucleotide variants (SNVs) between resistant and susceptible lines were identified using BCFtools (http://www.htslib.org/doc/bcftools.html) as described by Yemataw et al. (2018). The alignment results for the interval were visualized using Integrative Genomics Viewer (IGV) (Robinson et al. 2011) after converting to the BAM format (Li et al. 2009).

### Developing molecular markers

Before SNPs were converted into PCR-based Cleaved amplified polymorphic sequences (CAPS) markers, polymorphic sites were confirmed both on parents and bulks. We then randomly selected candidates to cover the 1.2 Mb region and the SNPs were converted into CAPS marker using dCAPS (http://helix.wustl.edu/dcaps/dcaps.html) (Neff et al. 2002). All PCR amplifications and digestion of PCR products with relevant restriction enzymes were performed by following manufacturers’ instructions and were visualized as described (Devran et al. 2015).

### Confirmation of linkage between established and newly generated markers

Newly generated PCR-based markers were first tested on parents to confirm the identified polymorphisms and then on a segregating 542 F_2_ lines. Marker genotyping data and the fungal disease phenotyping data were used to identify the *Frl* interval. Recombinant lines and the physical map covering the *Frl* region were used to narrow the interval for generation of new markers that could be used in the MAS-programme. Sequences of PCR-based markers will be provided upon request.

### Accession numbers

Tomato reference genome sequence (Tomato Genome Consortium 2012) GenBank: GCA_000188115.2 Solyc2.50. Accession number for the tomato chromosome 9 used is CM001072. The accession number for Sequenced Read Archive (SRA) is SRP138888.

## Results

### *Frl* segregates as a single locus

**Fig. 1:**
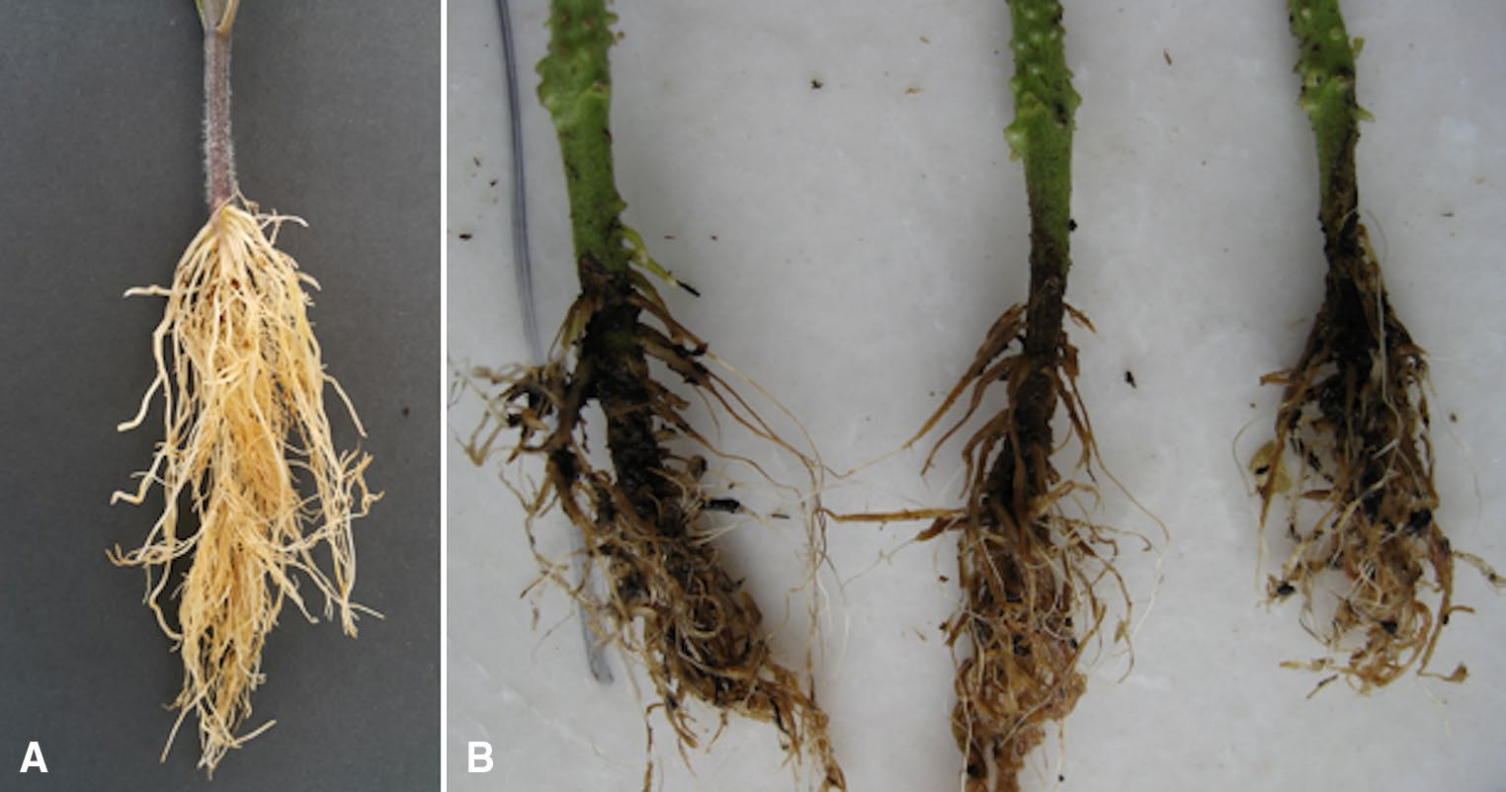
Healthy and *FORL*-infected tomato roots. The roots of seedlings at one true leaf stage were washed off substrate, dipped in a suspension of 10^7^ spores/ml. Seedlings were then transplanted in a sterilized mixture and kept in a growth chamber at 23 °C with a 12 h photoperiods for 30 days. Control plants were treated with sterile dH_2_0 in a similar manner. **a** Control roots, **b** *FORL*-infected roots

The susceptible *S. lycopersicum* pure line MT-7000 was crossed with the resistant, MT-7028, line. The F_1_ generation showed resistance to *FORL,* indicating that resistance was dominant. A population of 542 segregating F_2_ individuals derived from the F_1_ was then screened with the fungus. Disease symptoms, rotting in the tap roots, chocolate brown cankers appearing at the soil line in the susceptible plants, were clearly visible after 30 days (Fig. 1). The segregation ratio observed in this bioassay was 415:127 (resistant:susceptible, 3:1; with Chi square = 0.05 and P = 0.05). This suggested that a single resistance locus, *Frl*, was controlling the resistance in this cross, confirming the previous findings and allowing the subsequent analysis.

### Fine mapping defines a 900 kb interval for the *Frl* locus

**Fig. 2:**
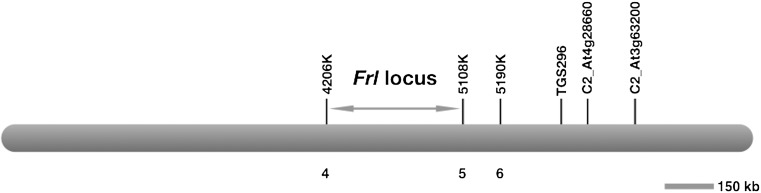
Map interval of *Frl* on tomato chromosome 9. Position of molecular markers used to map the *Frl* locus on the reference tomato genome. Numbers below the bar indicate the number of recombinants in 542 F_2_ individuals

DNA from twenty resistant and twenty susceptible F_2_ lines was pooled in equal concentrations to make up the resistant and susceptible bulks, respectively. We generated 100 bp paired-end Illumina HiSeq 2500 sequencing data from the two bulks (resistant and susceptible), comprising 46 million reads for the resistant and 42 million reads for the susceptible parent. Similarly, 80 million reads for the resistant parent and 118 million for the susceptible parent were generated. We then used the published tomato reference genome sequence (Tomato Genome Consortium 2012) (GenBank: GCA_000188115.2 Solyc2.50) as a reference to map sequence reads from both resistant and susceptible parents, and as well as sequences from both bulks. We concentrated on chromosome 9 around the previously published markers that were claimed to be linked to *Frl* (Truong et al. 2011; Mutlu et al. 2015). Initially, 1.2 Mb region covering some of the previous markers was taken into account and some of the identified SNVs at the flanking regions were converted to CAPS marker and were then used to map the *Frl* locus. Once linkage was confirmed (Table 1), a total of 542 F_2_ lines were then screened with further markers and the *Frl* locus was fine mapped to a 900 kb interval on the reference genome between the molecular markers 4206 and 5108 K (Fig. 2). A gel image of segregating F_2_ lines was given as a representative of mapping *Frl* (Supplementary Figure 1).

**Table 1:** Segregation of locus among F_2_ lines that were critical to the mapping of *Frl*

*Frl* interval on chromosome 9							
F_2_ Lines*	4206 K	4876 K	4942 K	*Frl*	5023 K	5108 K	5190 K
26	SS	SS	SS	SS	SS	**RS**	**RS**
220	SS	SS	SS	SS	SS	**RS**	**RS**
247	RS	RS	RS	R	RS	**SS**	**SS**
261	RS	RS	RS	R	RS	RS	**RR**
321	**RS**	SS	SS	SS	SS	SS	SS
349	SS	SS	SS	SS	SS	RS	RS
377	**RS**	SS	SS	SS	SS	SS	SS
439	**RS**	SS	SS	SS	SS	SS	SS
560	**RS**	SS	SS	SS	SS	SS	SS
518	SS	SS	SS	SS	SS	RS	RS

*F_2_ lines were generated from the cross between the resistant and the susceptible cultivars. *SS* homozygous for susceptible parent allele; *RR* homozygous for resistant parent allele; *RS* heterozygous. Important recombinants are given in bold

### Interval contains single-nucleotide variants

It is imperative to develop MAS-friendly markers that are very tightly linked to the gene of interest (Foolad et al. 2008) in order to carry out high-throughput breeding programmes. As the number of F_2_ lines used was 542, it is expected that the number of lines showing recombination event would not be high enough in the tomato genome to narrow the genetic and physical interval. Although we have defined the interval to a 900 kb region, the 900 kb size of the interval means that there is the possibility of internal recombination events unlinking the marker from the causal locus, which may prove difficult to carry out large-scale breeding programmes. Therefore, using the NGS data from parents and the bulks, we mined the data for single-nucleotide polymorphisms (SNPs) in an attempt to identify the locus with finer resolution. We examined only the 900-kb interval region and a total of 1099 single-nucleotide sites found to be polymorphic (Supplementary Table 1). A heatmap for the polymorphisms was produced (Supplementary Figure 2). We converted three of these single-nucleotide polymorphisms (SNPs) to CAPS markers, 4876, 4942 and 5023 K, and used them to map *Frl*. They all co-segregate with *Frl* (Table 1), confirming the genetic interval and the molecular polymorphism.

### Marker co-segregating with *Frl* differentiates commercial varieties

The ultimate aim of any marker development effort is effective use in breeding programmes that include backcrossing, gene pyramiding, eliminating unsuitable lines and accelerated line developments (Collard and Mackill 2008). With this in mind, we have evaluated our newly developed markers against 50 commercial tomato varieties, 24 of which are with known *Frl* phenotypes, obtained as seeds. We grew them to seedlings and isolated DNA. We then performed PCR with co-segregating molecular markers. One of our markers was polymorphic with all the F_1_s and co-segregated with the claimed phenotypes. Representatives of these varieties and their claimed phenotype and the *Frl* genotype with the marker are given in Table 2. This indicates that the identified polymorphism has been maintained across different varieties during independent breeding programmes and, thus, the marker is tightly associated with *FORL* resistance and may be close enough to the causal genetic element for resistance to be useful in breeding programmes.

**Table 2:** Phenotype of commercial tomato varieties and their genotype with the *Frl* marker developed in this study

Commercial variety^a^	Claimed phenotype^b^	Genotype with *Frl* marker
Alberty F_1_	Resistant	RS
Corvette F_1_	Resistant	RS
Avalantino F_1_	Resistant	RS
Alyanak F_1_	Resistant	RS
Akın F1	Resistant	RS
Çikoköy F_1_	Resistant	RS
Seval F_1_	Resistant	RS
Vertigo F_1_	Susceptible	SS
Moda F_1_	Susceptible	SS
Pony Express F_1_	Susceptible	SS

^a^These are selected from 50 readily available varieties on the market

^b^Phenotype information has been obtained from the companies’ websites, which sell these varieties to growers

*RS* resistant/susceptible heterozygous, *SS* susceptible homozygous

### The *Frl* interval contains defense-related genes

We attempted to identify gene candidates that could be causally linked to the resistance phenotype, using the annotations of the tomato reference genome (Tomato Genome Consortium 2012). The *Frl* interval contains a total of 107 predicted genes according to the International Tomato Annotation Group (ITAG) version 3.2 (Supplementary Table 2). Seven of these genes have been annotated as glutathione S-transferase, nine of them as glutathione S-transferase-like proteins and two of them as putative glutathione S-transferase T2 proteins. Interestingly, these 18 genes (Table 3) are clustered in the centre of the interval as a gene family around the co-segregating marker. In addition to these genes, the interval also contains other defence-related genes including lectin receptor kinase, leucine-rich repeat-containing protein, serine/threonine-protein kinase and kinase family protein (Table 3).

**Table 3:** Defence-related genes within *Frl* interval

Gene ID	Putative function
Solyc09g011027.1.1	Pathogenesis-related thaumatin family protein
Solyc09g011060.2.1	Clade IV lectin receptor kinase
Solyc09g011070.1.1	clade XI lectin receptor kinase
Solyc09g011235.1.1	Leucine-rich repeat-containing protein
Solyc09g011320.3.1	Serine/threonine-protein kinase
Solyc09g011330.2.1	Serine/threonine-protein kinase
Solyc09g011490.3.1	Glutathione S-transferase-like protein
Solyc09g011500.3.1	Glutathione S-transferase-like protein
Solyc09g011510.2.1	Glutathione S-transferase-like protein
Solyc09g011520.3.1	Glutathione S-transferase-like protein
Solyc09g011530.2.1	Glutathione S-transferase-like protein
Solyc09g011535.1.1	Glutathione S-transferase-like protein
Solyc09g011540.2.1	Glutathione S-transferase
Solyc09g011550.2.1	Glutathione S-transferase
Solyc09g011560.2.1	Glutathione S-transferase
Solyc09g011570.3.1	Glutathione S-transferase-like protein
Solyc09g011580.2.1	Glutathione S-transferase-like protein
Solyc09g011590.3.1	Glutathione S-transferase-like protein
Solyc09g011600.3.1	Glutathione S-transferase
Solyc09g011610.3.1	Glutathione S-transferase
Solyc09g011620.1.1	Glutathione S-transferase
Solyc09g011630.3.1	putative glutathione S-transferase T2
Solyc09g011640.4.1	Putative glutathione S-transferase T2
Solyc09g011650.3.1	Glutathione S-transferase
Solyc09g011750.3.1	Kinase family protein

## Discussion

Tomato breeding efforts have been carried out since 1930s and as the technology in molecular biology developed, the use of molecular markers and genetic maps has contributed enormously to the tomato crop improvement (Foolad and Panthee 2012). Using the current NGS technology, we demonstrate evidence that it is possible to generate MAS-friendly markers tightly associated with *FORL resistance locus* (*Frl*) in tomato. The *Frl* locus was originally identified in a mutant of wild-type tomato *S. peruvianum* (also known as Peruvian nightshade). It has been introgressed into the cultivated tomato varieties (Foolad and Panthee 2012) and has been used in many breeding programmes. Previous mapping exercises have placed *Frl* on the chromosome 9 closely linked to the Tm-2^2^ gene (Vakalounakis et al. 1997). Several groups have used different approaches to generate PCR-based markers including RAPD (Fazio et al. 1999) and SCAR (Mutlu et al. 2015) markers. However, to our knowledge, the use of these markers in breeding programmes has not been reported. In addition, in our own research selection for *Frl* in the tomato breeding proved difficult as these markers were not reliable, most likely due to not being tightly linked to the causal locus. This prompted us to initiate the present investigations, where we generated a mapping population between a resistant and a susceptible parent and used it to identify the tightly associated markers. Bulk segregant analysis (Michelmore et al. 1991) for gene mapping has been used in many different crop species as well as in microbial pathogens. Although the technique was originally used with RAPD markers, since then, it has been widely used in combination with the NGS technology (Devran et al. 2015; Woods-Tör et al. 2018). We utilized this advantage and sequenced the genomic DNA from resistant and susceptible bulks as well as from the parents. We mapped the raw sequences onto the published tomato reference genome and identified SNPs. Subsequently, a few SNPs within 1.2 Mb region of *Frl* locus were converted into PCR-based CAPS markers and the *Frl* was fine mapped to a 900 kb interval using 542 F_2_ lines.

When interval is large, it is recommended that the selection of a trait is carried out with two flanking makers to maximize the probability of success (Collard and Mackill 2008). However, for large-scale breeding programmes, this may not be suitable as it becomes labour intensive. To overcome this and generate co-segregating markers, we searched the entire interval for SNVs and identified 1099 polymorphic sites. Generating PCR-based markers from some of these SNPs enabled us to develop new markers. As the generation of pure lines involves many crossings and selfings, the chances of a recombination event increase with each additional crossing and, thus, tight linkage of marker becomes more important (Yan et al. 2017). Testing our markers on commercial varieties that had been independently developed showed that these new markers also co-segregated with *Frl* phenotype, indicating that the marker generated in this study is very tightly linked to *Frl* and applicable to multiple varieties.

We showed that *Frl* interval contains defence-related genes (Table 3). It is well known that serine/threonine-protein kinase encoding genes such as *PTO* and *PTI* are involved in resistance to plant pathogens (Zhou et al. 1995). Similarly, lectin receptor kinase proteins have been reported to be involved in innate immunity in plants (Singh and Zimmerli 2013). Most intriguing was the presence of genes encoding the glutathione S-transferase (GST) family proteins within the interval. GSTs are abundant proteins encoded by a highly divergent ancient gene family and represent a major group of detoxification enzymes (Edwards et al. 2000). It is well known that during fungal infections, oxidative stress is induced and the GSTs contribute to the defence response (Gullner and Komives 2006). There were 18 genes as a cluster encoding them in the vicinity of the co-segregating markers. Interestingly, using a proteomic approach, Mazzeo et al. (2014) have reported the increased level of a GST-like protein, Solyc09g011590 in *FORL*-resistant tomato plant. It is tempting to speculate that some of these GSTs may be responsible for *FORL* resistance.

## Electronic supplementary material

Below is the link to the electronic supplementary material.

A gel image of segregating F_2_ lines as a representative of mapping *Frl*. DNA isolated from F2 lines were PCR amplified using the primers for the marker 5023 and digested with the relevant enzyme. The products were then run on a 1% gel and visualized under UV light after staining with ethidium bromide. M, Size marker; P1, Parent 1, P2, Parent 2 (TIFF 6395 kb)

Heat-map of estimated allele frequencies across 1098 single-nucleotide polymorphisms on the interval on tomato chromosome 9. The colours indicate the proportion of reads matching the variant allele such that yellow indicates all reads match the reference genome and red indicates all reads divergent from the reference genome. Orange indicates a mixture of matching and divergent reads; in the parental genomes this indicates heterozygosity whereas in the bulk populations it indicates heterozygosity and/or intra-population variation. Each column represents one of 1098 single-nucleotide sites identified by aligning genomic sequence reads against the reference genome (GenBank: AEKE00000000.2) using BWA (Li and Durbin 2009) and calling SNPs using the Sequence Alignment/Map tools (SAMtools)/binary call format tools (BCFtools) package (Li et al. 2009) as previously described (Yemataw et al. 2018). Columns are ordered according to their physical position on chromosome 9 (GenBank: CM001072.2) and fall between positions 4,207,531 and 5107918 (TIFF 7733 kb)

SNPs and their positions in *Frl* interval on chromosome 9 (XLSX 57 kb)

Genes within the *Frl* interval and their putative function (XLSX 13 kb)
