# Supplementary figures and images for: Identifying molecular markers suitable for Frl selection in tomato breeding
Source: Theor Appl Genet. 2018 Jul 7;131(10):2099–105. doi: 10.1007/s00122-018-3136-0 (PMC6154021; doi:10.1007/s00122-018-3136-0)

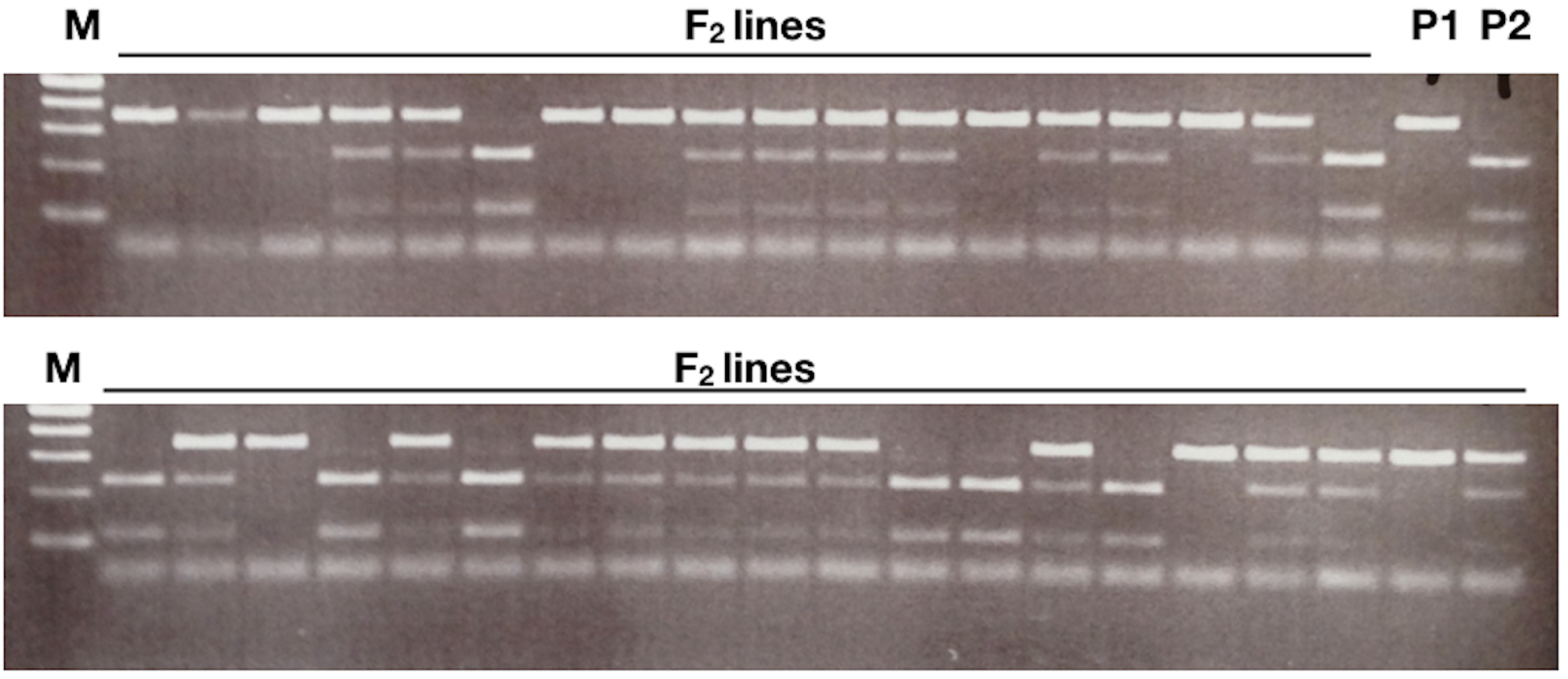

Supplement: Supplementary file 1 — A gel image of segregating F2 lines as a representative of mapping Frl. DNA isolated from F2 lines were PCR amplified using the primers for the marker 5023 and digested with the relevant enzyme. The products were then run on a 1% gel and visualized under UV light after staining with ethidium bromide. M, Size marker; P1, Parent 1, P2, Parent 2 (TIFF 6395 kb) [file 122_2018_3136_MOESM1_ESM.tiff]

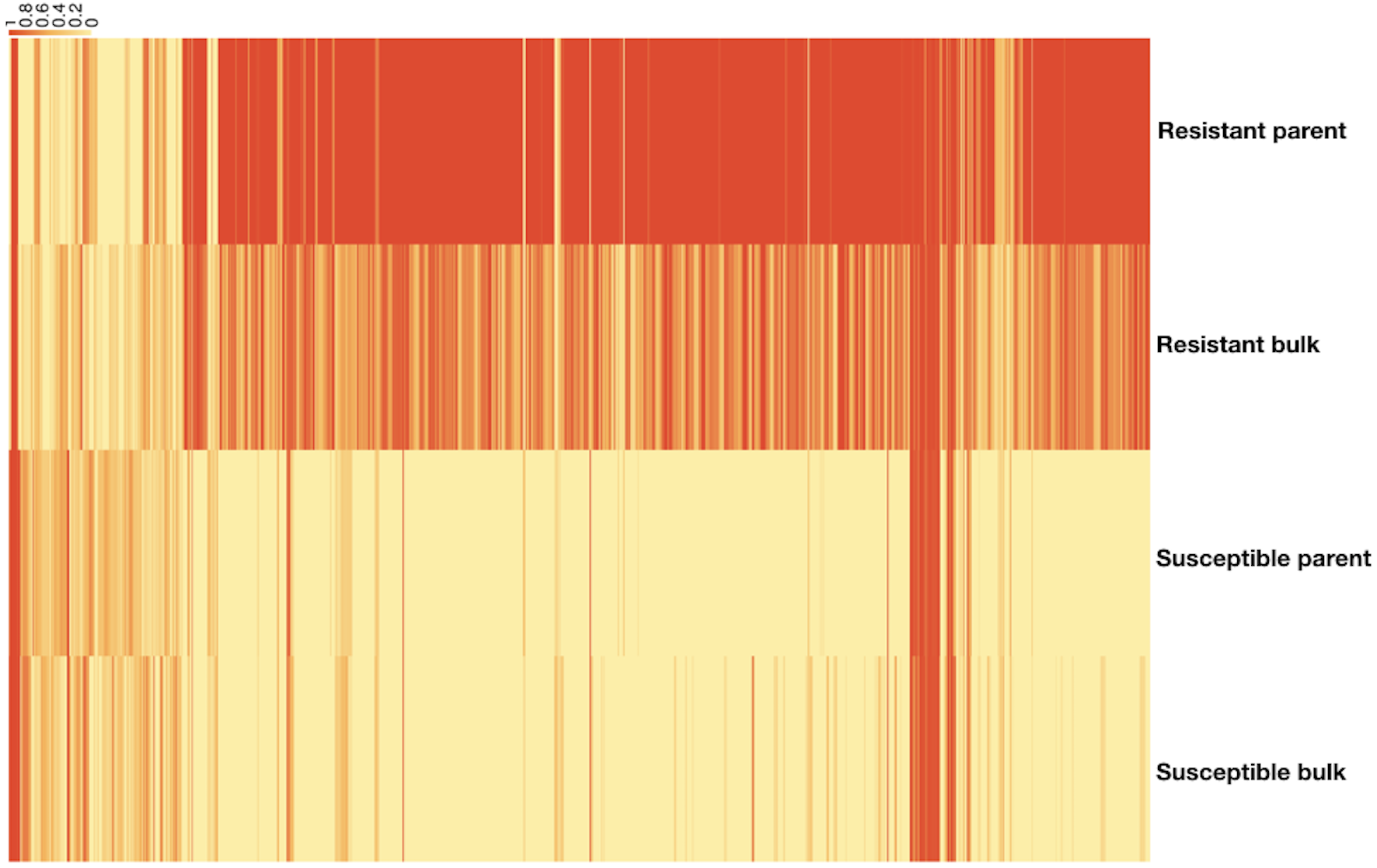

Supplement: Supplementary file 2 — Heat-map of estimated allele frequencies across 1098 single-nucleotide polymorphisms on the interval on tomato chromosome 9. The colours indicate the proportion of reads matching the variant allele such that yellow indicates all reads match the reference genome and red indicates all reads divergent from the reference genome. Orange indicates a mixture of matching and divergent reads; in the parental genomes this indicates heterozygosity whereas in the bulk populations it indicates heterozygosity and/or intra-population variation. Each column represents one of 1098 single-nucleotide sites identified by aligning genomic sequence reads against the reference genome (GenBank: AEKE00000000.2) using BWA (Li and Durbin 2009) and calling SNPs using the Sequence Alignment/Map tools (SAMtools)/binary call format tools (BCFtools) package (Li et al. 2009) as previously described (Yemataw et al. 2018). Columns are ordered according to their physical position on chromosome 9 (GenBank: CM001072.2) and fall between positions 4,207,531 and 5107918 (TIFF 7733 kb) [file 122_2018_3136_MOESM2_ESM.tiff]
